# Influence of a diet enriched with virgin olive oil or butter on mouse gut microbiota and its correlation to physiological and biochemical parameters related to metabolic syndrome

**DOI:** 10.1371/journal.pone.0190368

**Published:** 2018-01-02

**Authors:** Isabel Prieto, Marina Hidalgo, Ana Belén Segarra, Ana María Martínez-Rodríguez, Antonio Cobo, Manuel Ramírez, Hikmate Abriouel, Antonio Gálvez, Magdalena Martínez-Cañamero

**Affiliations:** 1 Área de Fisiología, Departamento de Ciencias de la Salud, Universidad de Jaén, Jaén, Spain; 2 Área de Microbiología, Departamento de Ciencias de la Salud, Universidad de Jaén, Jaén, Spain; 3 Departamento de Estadística e Investigación Operativa, Universidad de Jaén, Jaén, Spain; INIA, SPAIN

## Abstract

The type of fat in the diet determinates the characteristics of gut microbiota, exerting a major role in the development of metabolic syndrome. We hypothesize that a diet enriched with extra virgin olive oil (EVOO) has a distinctive effect on the intestinal microbiome in comparison with an enriched butter diet (BT) and this effect is related to the physiological benefits exerted by EVOO. Swiss Webster mice were fed standard (SD) or two high fat diets enriched with EVOO or butter. Hormonal, physiological and metabolic parameters were evaluated. At the end of the feeding period, DNA was extracted from faeces and the 16S rRNA genes were pyrosequenced. Among the main significant differences found, BT triggered the highest values of systolic blood pressure, correlating positively with the percentage of *Desulfovibrio* sequences in faeces, which in turn showed significantly higher values in BT than in EVOO. EVOO had the lowest values of plasmatic insulin, correlating inversely with *Desulfovibrio*, and had the lowest plasmatic values of leptin which correlated inversely with *Sutterellaceae*, *Marispirillum* and *Mucilaginibacter dageonensis*, the three showing significantly higher percentages in EVOO. The lowest total cholesterol levels in plasma were detected in SD, correlating positively with *Prevotella* and *Fusicatenibacter*, both taxa with significantly greater presence in SD. These results may be indicative of a link between specific diets, certain physiological parameters and the prevalence of some taxa, supporting the possibility that in some of the proposed effects of virgin olive oil the modulation of intestinal microbiota could be involved.

## Introduction

Dietary fat intake determines the fatty acid composition of cell membranes and plays a well-recognized role in cardiovascular risk and the development of cardio-metabolic diseases such as those included in the concept of metabolic syndrome [1]. Diet has a marked influence on the intestinal microbiota, generating different enterotypes [2] with a predictable composition [3] according to the type of diet consumed. In particular, it has been clearly demonstrated that dietary fat intake is a major factor influencing the pattern of gut microbiota [4], and the *Bacteroidetes*/*Firmicutes* ratio [5]. Within this field, there is a marked interest on high fat diets due to their reported negative effects on health, mainly through its link to metabolic syndrome [6]. Increasing evidence suggests that the pattern of gut microbiota affects the body energy balance [7] leading significantly to the development of obesity, obesity-associated inflammation and insulin resistance, all of them factors involved in the metabolic syndrome [8–10]. Compared with diets enriched with saturated fatty acids, diets with a high percentage of unsaturated fatty acids (ie. olive oil) have been associated with a lower stimulatory effect on weight gain and hepatic lipid accumulation. These effects were related, using a phylogenetic microarray, to the diet-induced changes observed in gut microbiota [11]. There is a general agreement on relating the regular consumption of virgin olive oil in the diet with a lower incidence of metabolic syndrome [12]. These beneficial properties have been attributed not only to the fatty acid composition but also to the unsaponifiable matter, mainly the polyphenol content [13]. Virgin olive oil is widely used as the main fat at the core of Mediterranean Diet, and it has been suggested to be beneficial in the management of diseases involving chronic inflammation, such as metabolic syndrome [14]. However, in spite of this evidence and proposal, the link between virgin olive oil, gut microbiota and the different factors that constitute the metabolic syndrome has never been demonstrated.

Therefore, the objective of the present study was to compare the resulting gut microbiota, evaluated by pyrosequencing the 16S rRNA genes, and several hormonal, physiologic and metabolic parameters, their alteration being reported as risk factors for the development of metabolic syndrome, after a period of twelve weeks in which adult male mice were fed a standard diet (SD) and diets supplemented with differently saturated types of fats such as butter (BT) and extra virgin olive oil (EVOO). Butter high fat diet was chosen as a representative of a Western Diet based on saturated fat in opposition to extra virgin olive oil high fat diet, based on monounsaturated fat and classically used in the Mediterranean Diet.

## Results

### Physiological parameters

As shown in Table 1, at the end of the experimental period, BT fed animals had significant higher body weight than the rest of groups (p<0.01) but with non-significant lowest food intake levels. Although systolic blood pressure of the EVOO diet showed the lowest value, it did not differ significantly from the SD group. In contrast, butter fed animals showed a significant increment with respect to the other two diets (p<0.01). Neither water intake nor diuresis showed significant differences, although BT diet had the lowest values in both variables. Mice fed the two high fat diets (EVOO and BT) had significantly higher levels of total cholesterol in plasma than those on SD (p<0.01). However, EVOO had lower levels of triglycerides than animals fed the two other diets, being this difference statistically significant with respect to butter (p<0.05). EVOO average value of HDL/LDL ratio was the highest, being significant with respect to the standard diet (p<0.05). No differences between groups were found in fasting glucose levels although plasma insulin had significantly lower values in EVOO with respect to the BT group (p<0.05). Close to significant lower levels of leptin were found in EVOO mice compared with SD and BT diets (p<0.067) but no differences for the levels of ghrelin were established between groups.

**Table 1:** Physiological and metabolic responses of mice after twelve weeks of diet. Given values are mean ± SEM.

	Standard Diet	EVOO Diet	Butter Diet	p
Food Intake (g/day)	3.70±0.65	3.74±0.39	2.76±0.38	n.s.
Water Intake (mL/day)	8.71±1.61	11.28±1.60	6.67±1.95	n.s
Diuresis (mL/day)	2.44±0.70	2.68±0.65	1.65±0.63	n.s.
Body Weight (g)	39.09±1.17	38.62±0.71	42.15±0.61	a**
Systolic Blood Pressure (mmHg)	161.71±11.83	148.11±5.94	190.50±8.53	a**
Plasma Leptin (pg/mL)	1929.63±437.86	949.89±230.36	1433.23±226.95	n.s
Plasma Ghrelin (pg/mL)	55.0±17.56	94.3±62.36	78.02±42.82	n.s.
Plasma Insulin (pg/mL)	1253.28± 201.32	685.71± 139.57	1518.44± 329.97	b*
Plasma Glucose (mg/100 mL)	194.13±17.31	175.33±18.95	192.0±17.2	n.s.
Plasma Triglycerides (mg/100 mL)	39.11±11.88	26.01±3.77	48.94±4.89	b*
Plasma Total Cholesterol (mg/100 mL)	49.28±10.53	84.57±9.98	98.67±9.98	c**
Plasma HDL/LDL Ratio	0.20±0.02	0.38±0.07	0.32±0.03	d*

a: Differences in butter *vs* standard and EVOO diets.

b: Differences in EVOO *vs* butter diet.

c: Differences in standard *vs* EVOO and butter diets.

d: Differences in EVOO *vs* standard diet.

* p<0.05

**p<0.01.

### Sequencing, taxa adscription, percentage comparison and correlations

After pyrosequencing the microbial diversity in the 26 faecal samples, the amount of reads was stable, obtaining a mean length between 497.12 and 528.8 nt (543.44 and 554.14 after trimming and filtering) and a total amount of 191.34 MB. A blast search was performed in order to associate an organism to each sequence obtained. Once we had the associations, the reads were filtered, analysed and grouped based on family, genera and species levels. In total, 276421 sequences were detected and classified into 8 phyla, 82 families, 134 genera and 308 species.

**Fig 1:**
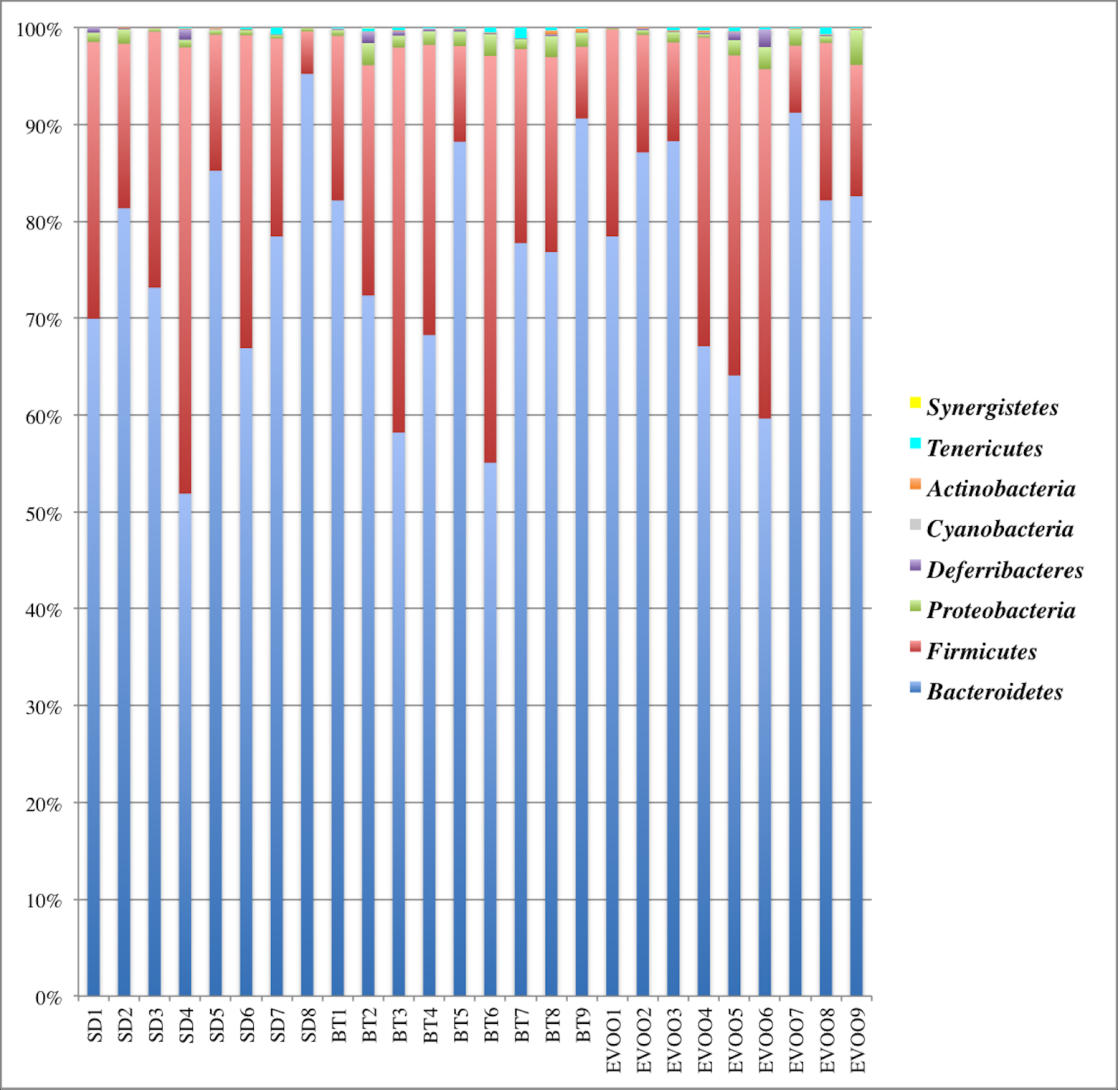
Global bacterial phyla distribution for each diet as a percentage of total sequences retrieved from faecal samples. Each column corresponds to one animal fed a diet enriched with butter (BT), virgin olive oil (EVOO) or standard chow (SD). Legend shows upwards the eight phyla detected.

**Fig 2:**
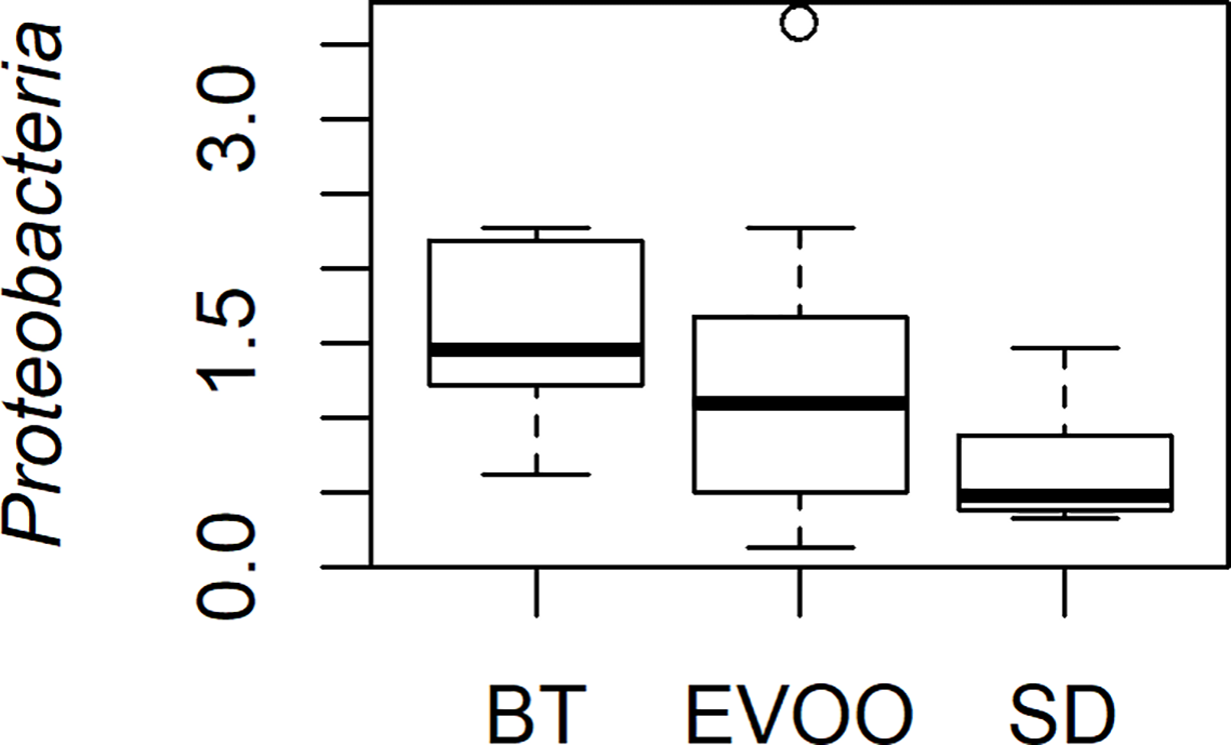
Box-plot representation of the percentage distribution of phylum *Proteobacteria* in faecal samples from mice fed a diet enriched with butter (BT), virgin olive oil (EVOO) or standard chow (SD). p value of pairwise comparison BT *vs* SD is 0.011.

Kruskal-Wallis test was used to check if the distributions of the diverse phyla were the same among the three diets (Fig 1). Only the phylum *Proteobacteria* showed significant differences (p = 0.039). Pairwise comparisons were performed showing that there are significant differences between BT and SD diets (p = 0.011) with a higher average level in the BT diet. Fig 2 shows the box-plot of the distribution of this phylum in the three diets. A linear regression analysis was performed for each physiological variable considering *Proteobacteria* as an independent variable. Conforming to this, the phylum *Proteobacteria* only showed correlation with ghrelin (R^2^ = 0.32; p = 0.0037).

**Fig 3:**
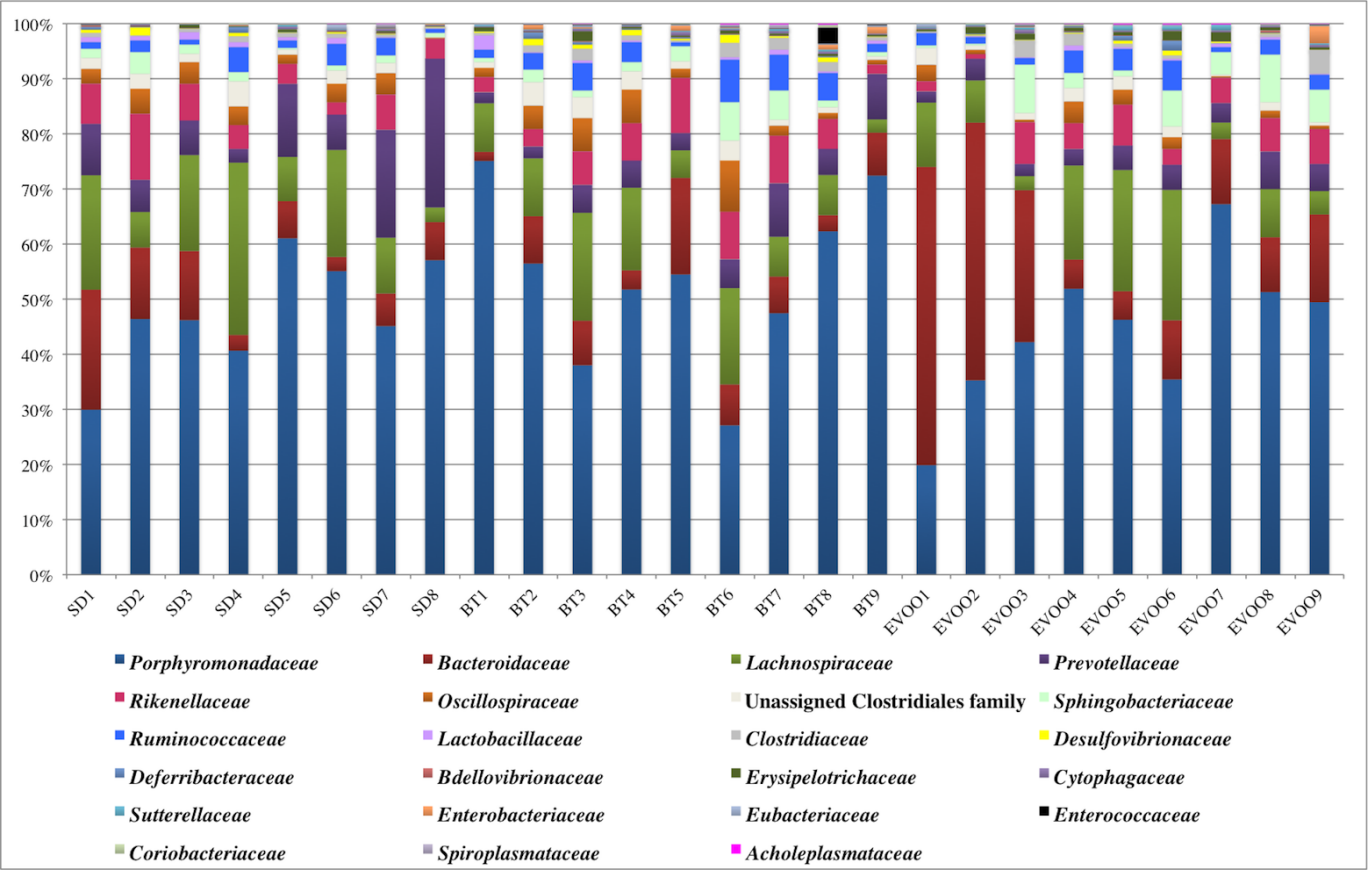
Bacterial family distribution as a percentage of total sequences retrieved from faecal samples. Each column corresponds to one animal fed a diet enriched with butter (BT), virgin olive oil (EVOO) or standard chow (SD). Legend shows, from left to right and downwards, the colour code of the 23 most prominent families.

**Fig 4:**
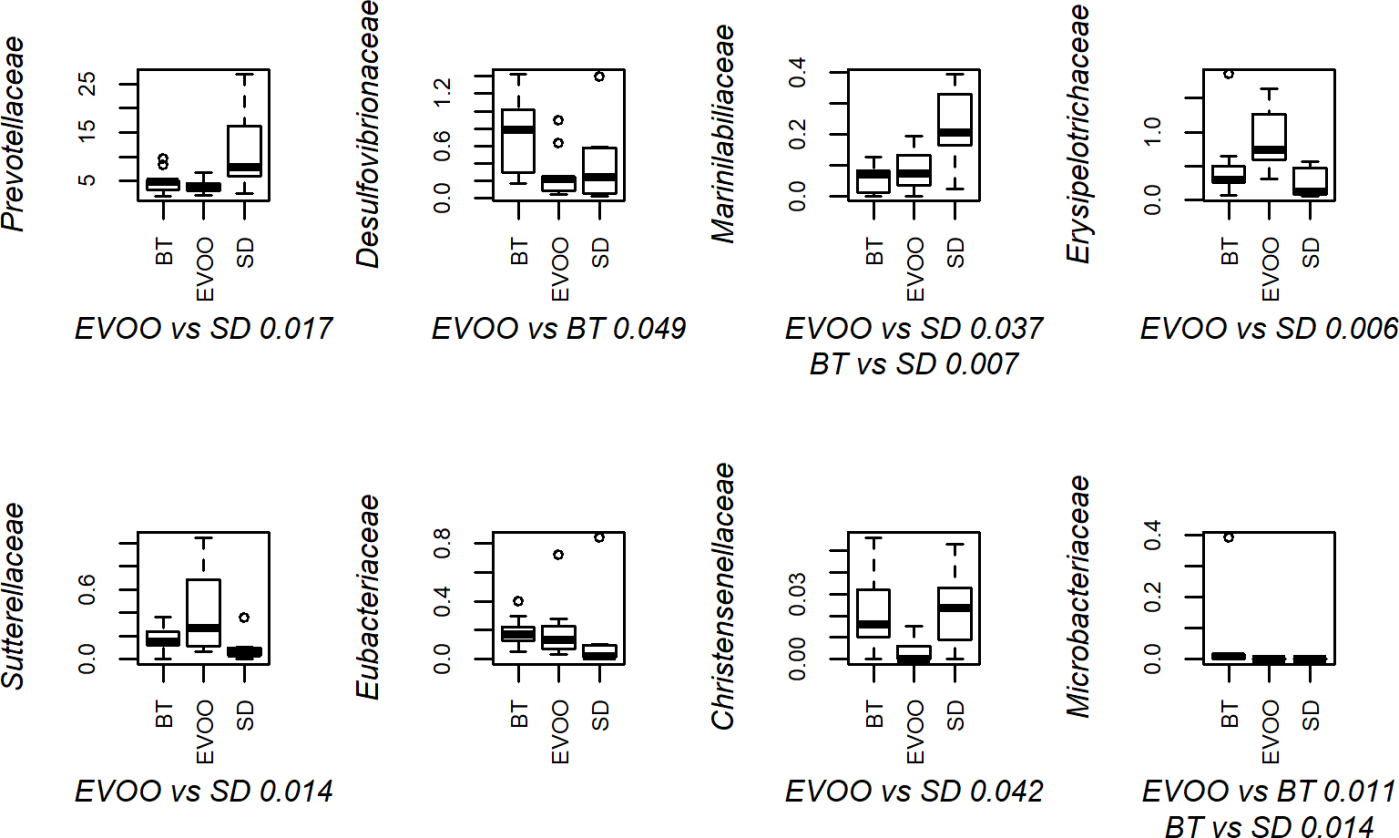
Box-plot representation of the percentage distribution of the eight families with significant differences (p<0.05) among faecal samples from mice fed a diet enriched with butter (BT), virgin olive oil (EVOO) or standard chow (SD). p values of significant pairwise comparisons are also shown.

According to the Kruskal-Wallis test, out of the 82 families that were detected (Fig 3), only eight of them showed statistical significant differences among the three diets. Fig 4 displays the box-plots of these eight families with the significant pairwise comparisons between diets, while all the pairwise comparisons and its p-values are summarized in S1 Table. Different multiple linear regression models were fitted to explain each physiological variable using as independent variables all the families with significant differences. Only total cholesterol, leptin and insulin values correlated significantly with four different families (Table 2).

**Table 2:** Regression fits for each of the physiological variables studied using as independent variables those families that show statistical differences in the percentage of sequences retrieved from faecal samples.

	TOTAL CHOLESTEROL (0.17/ 0.037)	LEPTIN (0.51/0.002)	INSULIN (0.19/0.025)
*Desulfovibrionaceae*			769.541± 321.79 (0.025)
*Erysipelotrichaceae*	28.7143±13.012 (0.037)		
*Sutterellaceae*		-1199.87±532.24 (0.035)	
*Eubacteriaceae*		2806.03±805.66 (0.002)	

For each case, regression coefficient estimate, s.e. and p values are shown. R^2^ and p values of the model are also indicated under each physiological variable.

**Fig 5:**
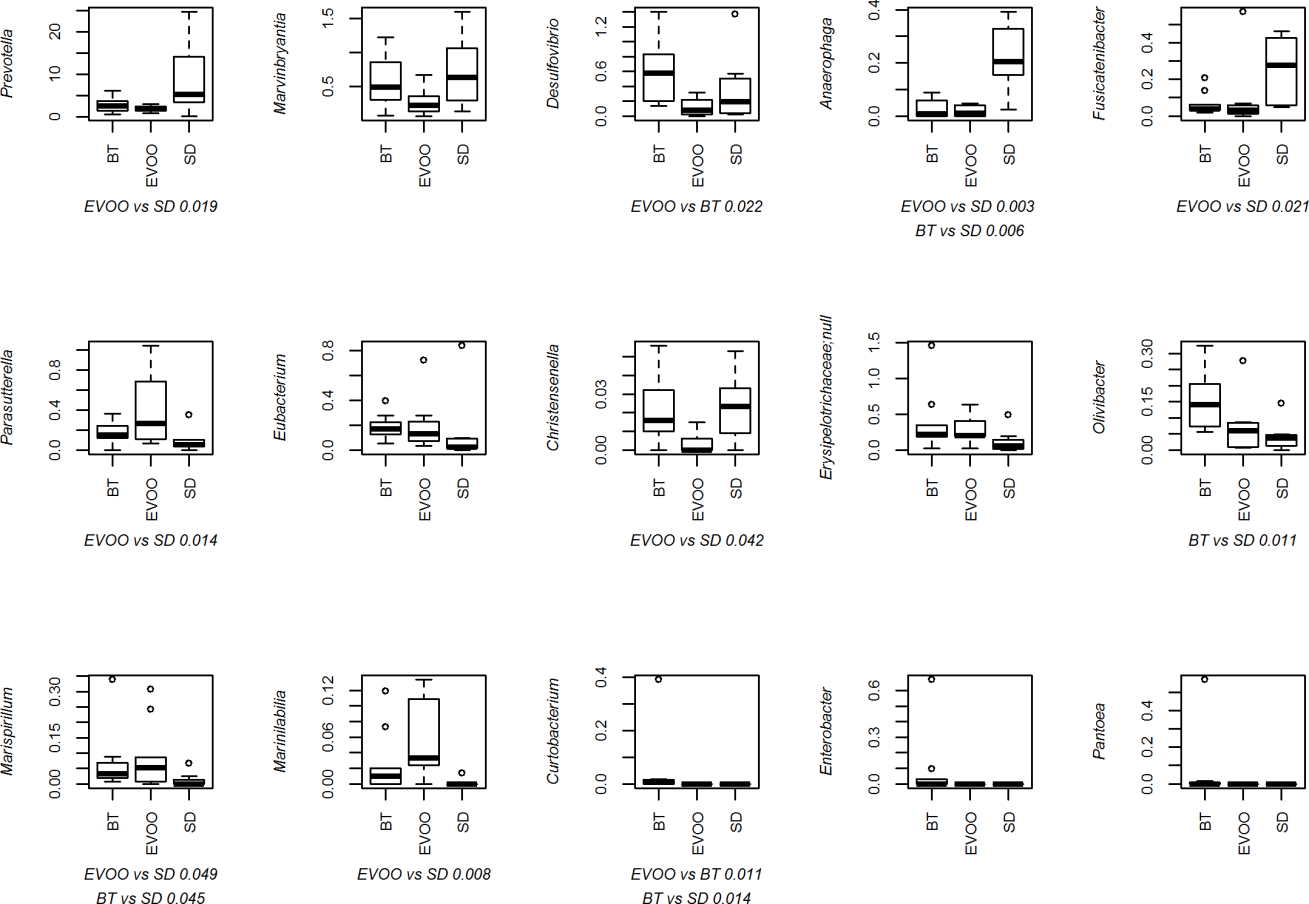
Box-plot representation of the percentage distribution of the fifteen genera with significant differences (p<0.05) among faecal samples from mice fed a diet enriched with butter (BT), virgin olive oil (EVOO) or standard chow (SD). p values of significant pairwise comparisons are also shown.

We also studied the percentages of the 134 genera obtained when studying the three diets. In this case, the Kruskal-Wallis test results (S2 Table) indicated that fifteen of them had significant differences (“*Erysipelotrichaceae*; null” indicates a group of genera framed into the *Erysipelotrichaceae* family that failed to match any described genus). Fig 5 shows the box-plots of all of these, including the significant pairwise comparisons between diets. Again, Table 3 shows the results found after applying a multiple linear regression analysis. Water intake, HDL/LDL ratio, total cholesterol, blood pressure, leptin, ghrelin, insulin and diuresis correlated significantly with at least one of eleven different genera.

**Table 3:** Regression fits for each of the physiological variables studied using as independent variables those genera that show statistical differences in the percentage of sequences retrieved from faecal samples. WI, water intake; T-CHO, total cholesterol; BP, blood pressure.

Genera	WI (0.28/0.042)	HDL/LDL (0.40/0.0027)	T-CHO (0.76/0.0001)	BP (0.31/0.004)	Leptin (0.69/0.0001)	Ghrelin (0.49/0.0008)	Insulin (0.18/0.026)	Diuresis (0.39/0.003)
*Prevotella*			-1.99108 ±0.8746 (0.0352)					
*Marvinbryantia*					969.032 ±334.16 (0.0091)			
*Desulfovibrio*	5.50586 ±2.4011 (0.0334)			41.4553 ±12.943 (0.0041)			843.087 ±357.66 (0.0269)	2.55017 ±0.7578 (0.0034)
*Anaerophaga*			129.75 ±46.7932 (0.0125)					
*Fusicatenibacter*		-0.50745 ±0.1560 (0.0035)	-142.861 ±27.701 (0.00007)					
*Parasutterella*		-0.24573 ±0.1005 (0.0226)	37.612 ±16.99 (0.0400)					
*Eubacterium*					2776.47 ±704.64 (0.0008)			
*Erysipelotrichaceae;* *null*			106.045 ±21.376 (0.0001)			-165.001 ±67.070 (0.0226)		
*Olivibacter*			263.0002 ±80.6559 (0.0043)			1060.19 ±240.76 (0.0002)		
*Marispirillum*					-3535.96 ±1234.9 (0.0099)			
*Enterobacter*	-14.2943 ±6.7884 (0.0487)							

For each case, regression coefficient estimate, s.e. and p values are shown. R^2^ and p values of the model are also indicated under each physiological variable.

**Fig 6:**
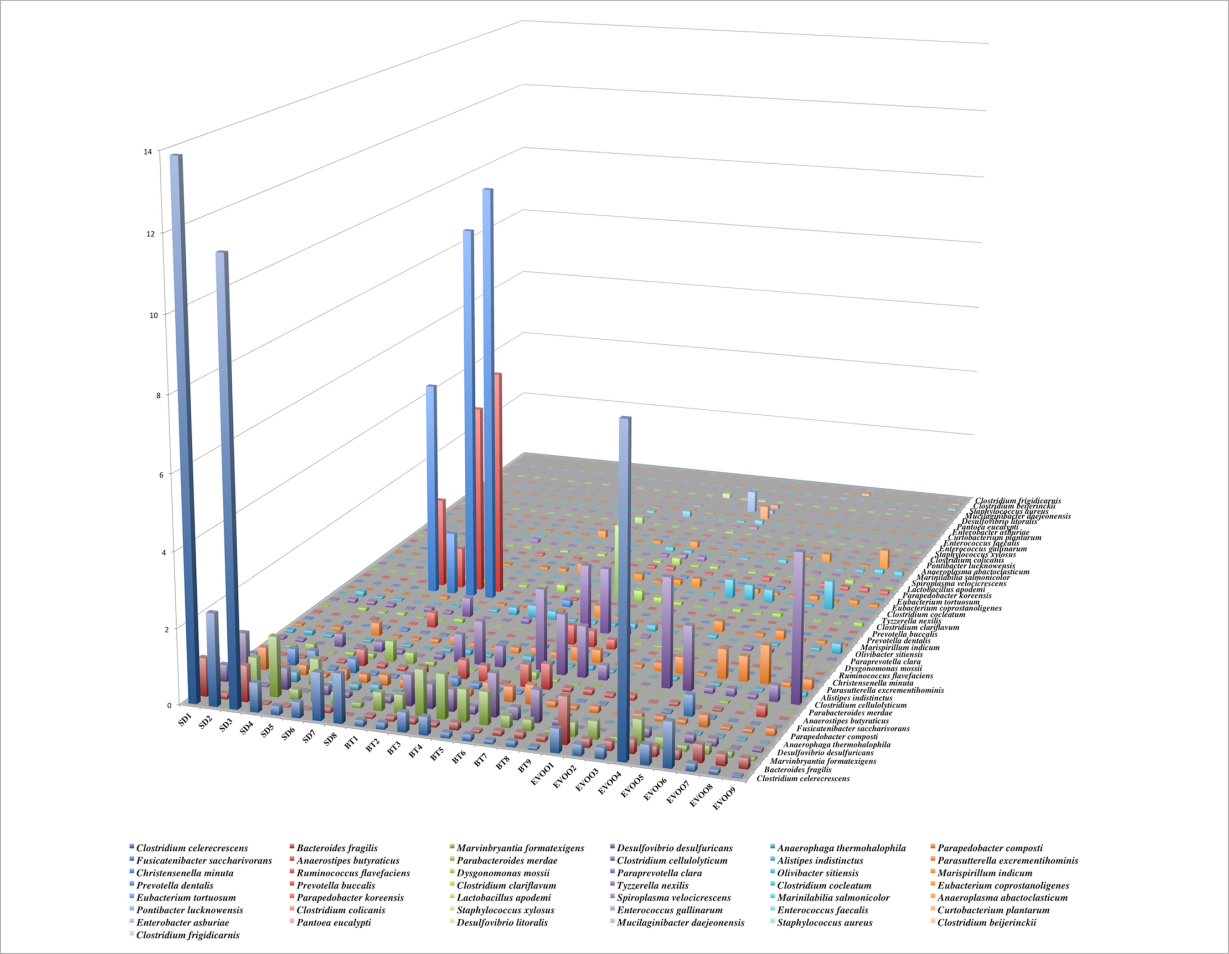
Distribution, as a percentage of total sequences retrieved from faecal samples, of the 43 species with significant differences between diets. Each column corresponds to one animal fed a standard chow (SD), enriched with butter (BT) or virgin olive oil (EVOO). Legend shows, from left to right and downwards, the colour code of these 43 species.

**Fig 7:**
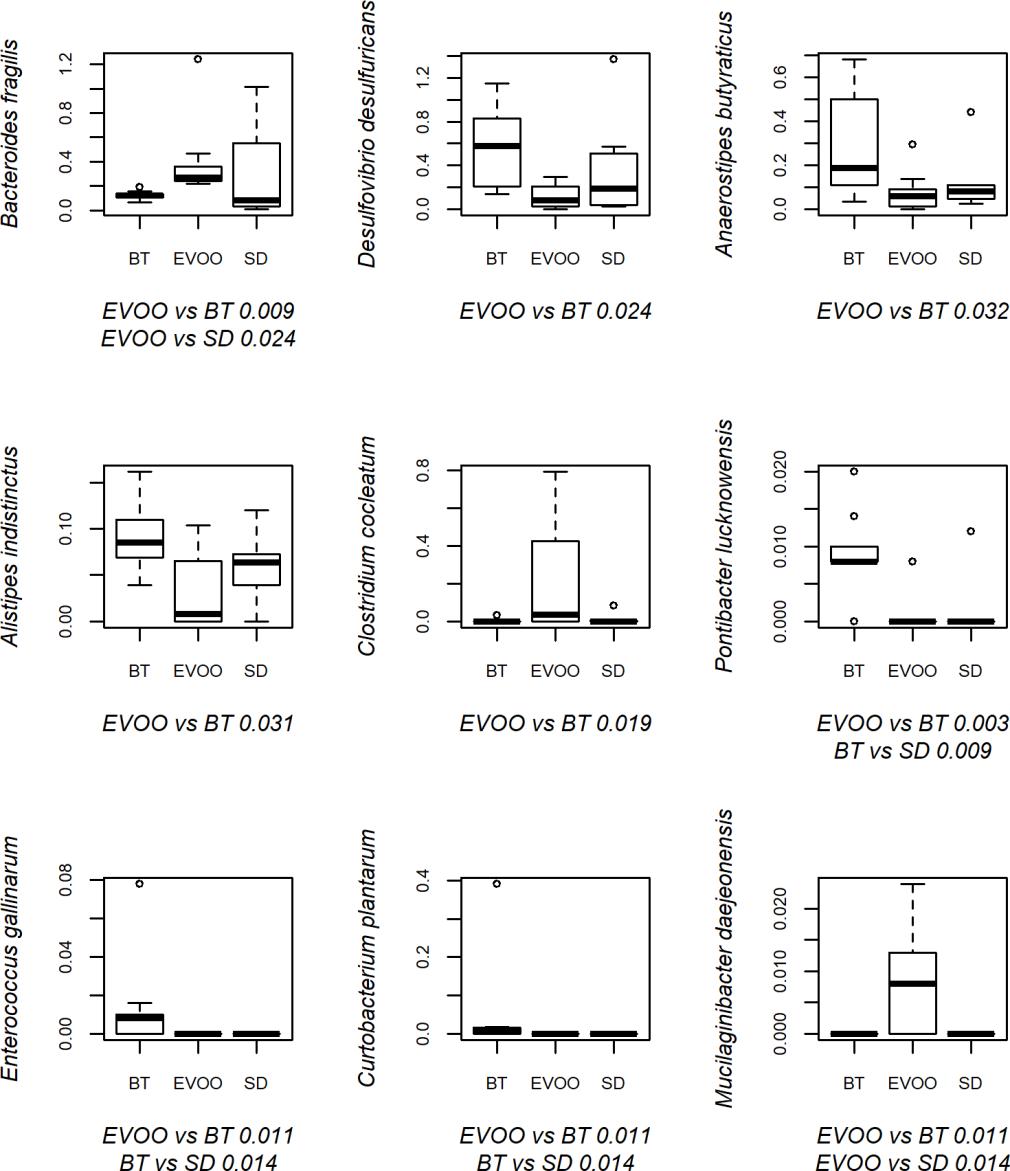
Box-plot representation of the percentage distribution of the nine species with significant differences (p<0.05) between faecal samples from mice fed a diet enriched with butter (BT) and a diet enriched in virgin olive oil (EVOO). p values of significant pairwise comparisons are also shown. SD, standard chow.

Finally, sequences were also analysed at species level in an indicative approach as explained below. When applying the Kruskal-Wallis test, 43 species showed significant differences as displayed in Fig 6, with statistical results shown in S3 Table. Due to this high number of species, which made impossible a multiple regression analysis that included all of them, and since our main interest was to highlight the differences between the results obtained comparing the EVOO and the BT diets, we decided to focus our work on those cases in which the EVOO-BT pairwise comparisons showed statistically significant differences. This reduced the number of species to nine, and their box-plot representation is shown in Fig 7 with the pairwise comparison between EVOO and BT groups. These nine species were used as independent variables in the regression analysis fitted for each physiological variable studied. In this case, *Curtobacterium plantarum* and *Enterococcus gallinarum* showed a high co-lineation coefficient so we performed the analysis twice with one of the two species each, resulting in very similar correlations. In Table 4, a summary of the analysis including *Enterococcus gallinarum* is shown. If *C*. *plantarum* was included instead, the same results were obtained except in the case of water intake, where no significant correlations were found then. When comparing *D*. *desulfuricans* with the results obtained in the correlations including the genus and family level (Tables 2 and 3), we considered that it could be interesting to check the relation of the species with the insulin plasmatic levels through a simple lineal regression, and when done, a coefficient of 970.672 (s.e. 376.304, p = 0.016 and R^2^ = 0.217) was obtained.

**Table 4:** Regression fits for each of the physiological variables studied using as independent variables those species that show significant differences between butter and virgin olive oil enriched diets in the percentage of sequences retrieved from faecal samples. FI, food intake; WI, water intake; BW, body weight; BP, blood pressure.

Species	FI (0.20/0.022)	WI (0.32/0.026)	BW (0.16/0.047)	HDL/LDL (0.25/0.036)	BP (0.33/0.003)	Leptin (0.17/0.046)	Insulin (0.30/0.003)	Diuresis (0.37/0.004)
*Bacteroides fragilis*	1.98546 ±0.8126 (0.022)			0.19888 ±0.0940 (0.045)				
*Mucilaginibacter daejeonensis*						-58479.4 ±27733.6 (0.046)		
*Alistipes indistintus*							9651.1 ±2973.26 (0.003)	
*Pontibacter lucknowensis*			192.067 ±91.7217 (0.047)	11.0151 ±5.1970 (0.045)				
*Enterococcus gallinarum*		-598.963 ±245.281 (0.025)						
*Desulfovibrio desulfuricans*		6.38154 ±2.5883 (0.023)			45.3297 ±13.748 (0.0032)			2.72514 ±0.8284 (0.0040)

For each case, regression coefficient estimate, s.e. and p values are shown. R^2^ and p values of the model are also indicated under each physiological variable.

Additionally, richness and diversity of taxa were estimated. Although the Shannon index did not reveal significant differences in the taxa diversity among the three groups at any level, we did obtain a positive outcome when analysing taxa richness after applying the Chao1 index, according to the Kruskal-Wallis test (p = 0,0029). The standard diet microbiomes showed a significantly different Chao 1 index value (average 133.5) when compared to EVOO and BT diets (average 204.5 and 226.1, respectively).

## Discussion

In this study, the profile of gut microbiota differed among diets enriched with different types of fats and specific microbial groups showed a significant correlation (with positive or negative character) with certain physiological and biochemical parameters directly or indirectly related to the metabolic syndrome.

Despite the similar energy content of both high fat diets, only the BT group developed the characteristic profile of metabolic syndrome. Although not significant differences between groups were achieved in food intake and even the mean value in the BT mice was the lowest, these animals showed significant higher body weight at the end of the experimental period. The effect of EVOO and, in particular, its main polyphenol, the hydroxythyrosol, have been reported to be beneficial in the prevention of metabolic syndrome [15], and recent results from our team also show a differential effect of diets enriched with virgin olive oil and butter on body weight and plasma lipid profile in wistar rats [16]. Systolic blood pressure values were also significantly increased in the BT group compared to the other two diets. In spite of this, there were not significant differences in water intake and diuresis values between the three groups. Virgin olive oil has demonstrated to be more efficient than other oils in reducing systolic and diastolic blood pressure in hypertensive patients [17] and in animal models of hypertension [18], which is in agreement with our present results. Regarding glycemic parameters, while no significant differences were found in fasting glucose levels, the amount of fasting insulin was significantly higher in BT than in the EVOO group.

**Fig 8:**
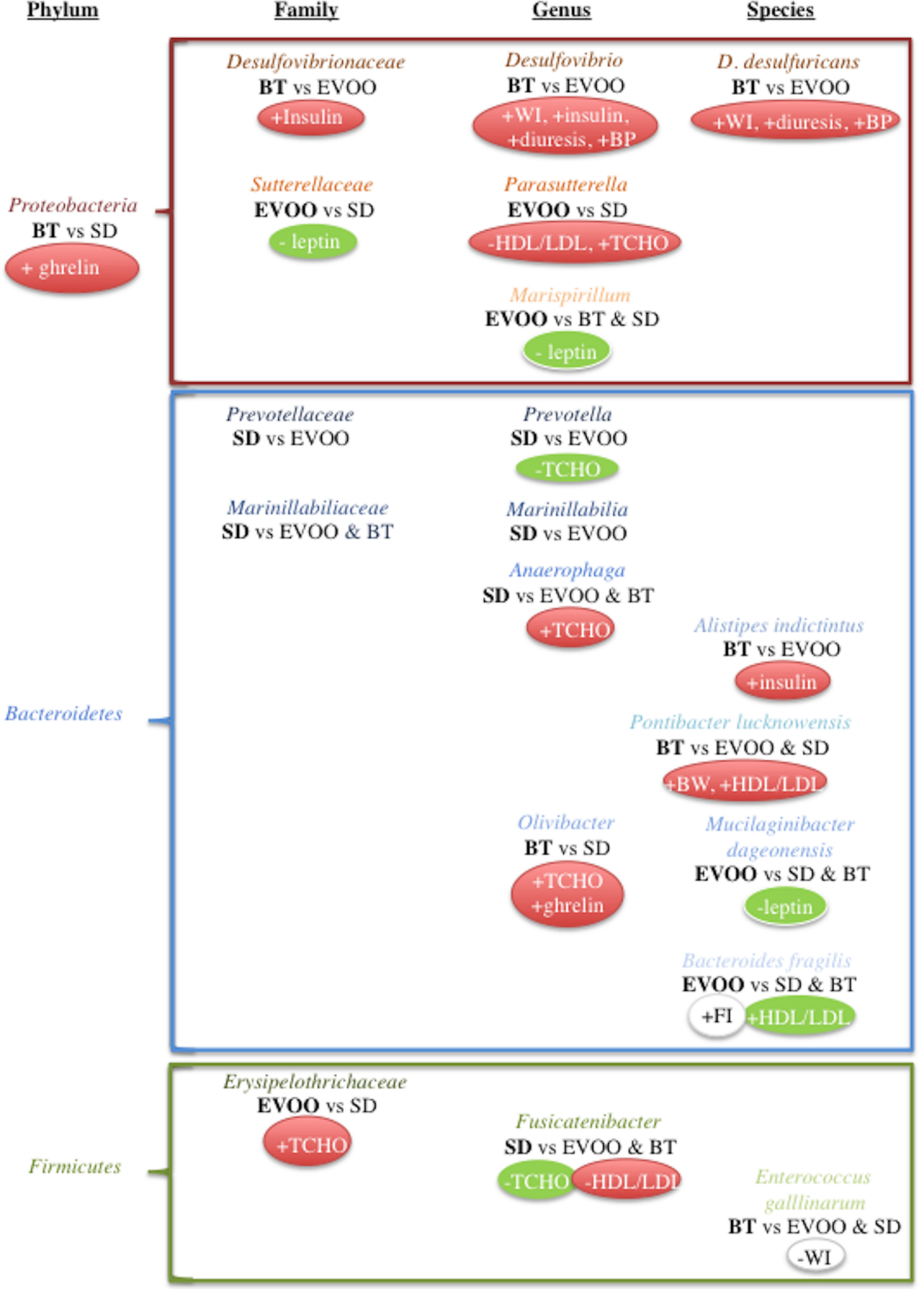
Summary of the statistically significant correlations between different physiological variables and taxa with statistically significant differences among diets as indicated by percentage of total assigned sequences retrieved from faecal samples. Bold letter indicates the diet where the taxon has higher presence. +, positive correlation; -, negative correlation. Red filling denotes outcomes that contribute to metabolic syndrome; green filling denotes outcomes that do not contribute to metabolic syndrome.

As a summary, Fig 8 grouped by phyla those taxa that show significant differences between groups and either correlate with the values of one or more physiological variables or include lower level taxa that fulfil these two requirements. One of the most remarkable results is the higher value of proteobacteria taxa observed in the BT diet. In fact, the average proteobacterial presence is elevated in both high fat diets as already described [19], but while these BT diet values are higher and statistically significant, in the case of EVOO diet they are lower on average and with no statistical significance. The phylum globally correlated with plasmatic ghrelin levels. This correlation did not appear again in the proteobacterial taxa that showed significant differences among the three diets, but it can be clearly (p<0.00001) observed in the case of the family *Enterobacteriaceae*, the genus *Escherichia* and the species *E*. *fergusoni*, all of them with a close-to-significant tendency to be higher in butter fed animals (p = 0.059, 0.070 and 0.070 respectively). *Proteobacteria* are Gram-negative bacteria, which are characterized by the presence of lipopolysaccharides (LPS) in their outer membrane. In this context, Chang *et al* [20] reported in rats that lipopolysaccharide stimulated directly gastric mucosa to synthesize and secrete ghrelin, which was considered by the authors to have a therapeutic effect on the endotoxic shock produced by LPS. Moreover, ghrelin was also shown to have antimicrobial activity against Gram-negative but not against Gram-positive bacteria [21], so it could be hypothesized that an increase in ghrelin secretion could be useful against an increment in LPS-bearing bacteria as it is the case with *Proteobacteria*. Notably, this relation is not shown in Table 1, where ghrelin levels do not have significant differences between diets, denoting that there may be other microbiota-independent factors affecting this hormone.

Another important difference between the two groups relies on which are the specific proteobacterial families that are increased in each one of the high fat diets. In the EVOO diet, only *Sutterellaceae* shows a significant increment, correlating inversely with leptin (together with *Marispirillum*, a *Rhodospirillaceae* genus), but this correlation is not maintained in the only significant genera included in this family, *Parasutterella*, which on the contrary correlates positively with total cholesterol and, inversely, with the ratio HDL/LDL. However, the diet enriched in butter showed a clear, significant increment in the presence of *Desulfovibrionaceae*, which was consistent with significantly higher levels of *Desulfovibrio* and its key species *D*. *desulfuricans*. In this respect, not only did the EVOO diet have significantly lower levels than those of the butter group but these values were also lower, although not significant, than those found in the standard diet at the three taxonomical levels. The presence of these taxa directly correlated with insulin plasmatic values (at the family and genus level) and with systolic blood pressure, water intake and diuresis (at the genus and species level). When tested, *D*. *desulfuricans* also showed a significant simple lineal regression with plasmatic insulin levels (p = 0.0164). Alterations in all these physiological variables are indicative of a metabolic syndrome condition. *Desulfovibrionaceae* are sulfate-reducing bacteria that could be sustained by butter sulfate sources, in part via cross-feeding mediated by *Bacteroides*-encoded sulfatases, since chondroitin sulfate, a common dietary supplement of animal origin, has been shown to stimulate *Desulfovibrio* intestinal growth [22]. Another *Desulfovibrionaceae*, *Bilophila wadsworthia*, has been shown to increase in high milk-derived fat diets as a consequence, according to the authors, of an increment in taurine-derived sulfur in the distal bowel [23]. The lower levels found in the EVOO diet, even with respect to the SD diet, could be explained by the elevated presence of polyphenols in this type of fat. In fact, another high polyphenol food, such as table grapes, has been shown to decrease the intestinal abundance of sulfidogenic *Desulfobacter* spp. and the *Bilophila wadsworthia*-specific dissimilatory sulfite reductase gene [24]. The correlations observed with *Desulfovibrio* are particularly interesting because this genus is the only one that correlates with BP and with other parameters also directly related to BP control such as WI and diuresis. The mechanism by which *Desulfovibrio* could be involved with BP control remains to be elucidated. However, since the levels of WI and diuresis did not differ between groups, it would be interesting to analyse if the predominance of these species would be related to an increase in cardiac output or peripheral vascular resistance as key factors in the control of BP. Although currently there is an intensive research on the role that dietary fats and gut microbiota play in the cardiovascular control [25], to our knowledge, there are still no reports that simultaneously connect type of diet, pattern of microbiota and cardiovascular control. Therefore, the present results strongly suggest for the first time a connection between these three factors.

*Desulfovibrio*, together with *Alistipes indictintus*, also correlates with plasmatic insulin levels, which are significantly higher in BT than in EVOO. In this sense, LPS-bearing bacteria have repeatedly been related to glucose intolerance; LPS-induced metabolic endotoxemia has been reported as the first step for the development of insulin resistance diabetes, and experimental LPS infusion has lead to fasted hyperglycemia and hyperinsulinemia [6]. Being part of the *Proteobacteria* and therefore Gram-negative bacteria, which present LPS in their outer membrane, *Desulfovibrio* fits this physiological effect. However in this study the correlation is not extended to the whole phylum neither to other proteobacterial taxa with significant differences between diets. Therefore, it is reasonable to consider other factors specific to this taxon affecting the LPS translocation through the tight junctions. One of these factors could rely on its sulfidogenic ability, since both sulphite and hydrogen sulphide have been implicated in intestinal toxicity and ultimately in the breakdown of the colonic epithelial cell barrier and the genesis of inflammation [26].

Plasmatic leptin values also show interesting results. Mice fed the EVOO diet present close to significant (p = 0.067) lower levels than BT and SD diets. The lower values obtained in EVOO versus BT are not surprising since leptin is a hormone secreted by fat cells that acts via hypothalamic receptors to inhibit feeding in order to reach energy balance and body weight regulation [27]. Therefore, higher levels are expected in the BT group since it has reached significantly higher average body weight than the other two groups. However, this is not the case with respect to the SD diet, where the average body weight is very similar to the EVOO group and thus, additional factors should be considered. In this regard, it is interesting to notice that in parallel we have found consistent inverse correlations of leptin plasmatic levels with bacterial taxa that are significantly increased in the EVOO group with respect to SD (*Sutterellaceae*, *Marispirillum* and *Mucilaginibacter dageonensis*, correlating these two latter ones also with BT). When comparing conventionally raised and germ-free mice, Schéle *et al* [28] found that gut microbiota reduces leptin sensitivity by increasing the hypothalamic expression of leptin resistance-associated suppressor of cytokine signaling 3. In a similar way, the EVOO diet could modify the gut microbiota structure, either directly activating bacterial groups that would inhibit this expression or, on the contrary, inhibiting the suppressor-inducing taxa and, consequently, promoting other taxa growth when leaving an empty niche that they are able to occupy.

Another difference between the microbial profile obtained with the EVOO diet and the one observed with the other two diets relied on the taxa within the *Bacteroidetes* phylum, which accounted for most of the sequences retrieved. We have not found global intergroup significant differences in the phylum *Bacteroidetes*. However, there was a clear tendency (p = 0.10) when Fam. *Bacteroidaceae* was considered (Fig 3), showing higher values in the EVOO group and lower in the other two groups (SD and BT). This pattern is also applicable to the genus *Bacteroides* (p = 0.10). On this subject, Patterson *et al*. [29] obtained significant differences both at the family and genus level when comparing palm, safflower, fish and olive oil with low fat diets, being olive oil the diet with the highest value. *Bacteroides* is a bile resistant strict anaerobe [30, 31] so this could be a reason for its specific increment after an olive oil diet, well known for its cholagogue effects [32], provoking a microenvironment that would give an adaptive advantage to the genus. In the same way, the presence of antioxidant polyphenols in virgin olive oil could protect anaerobic growth from oxygen microenvironments and this would explain as well the light increase of this genus in a safflower oil supplemented diet [29], also rich in polyphenols. Different *Bacteroides* species have been related to health benefits such as *B*. *uniformis* [33] or *B*. *fragilis* [34]. According to our present results, only one species, *B*. *fragilis*, has been shown to have a significant increase in EVOO diet versus the other two diets, probably because its lower percentage allowed less drastic intra-groups differences. This species correlated positively with the HDL/LDL ratio, a sign of cardiovascular health [35], which is a characteristic effect of olive oil based diets as shown in this work (Table 1) and others [36, 37].

In contrast to the EVOO diet, in the SD diet the phylum *Bacteroidetes* is clearly represented by the families *Prevotellaceae* and *Marinillabiliaceae*, showing a significant increase when SD and EVOO diets were compared. Genus *Prevotella* has a highly significant presence in SD diet, correlating inversely with total cholesterol, which accounts for the significant differences observed between SD and high fat diets and described in Table 1. *Prevotella* is related to plant-rich diets with high intake of carbohydrates and vegetables [3] and it has been related to an improvement in glucose metabolism [38] but its relation to cholesterol is not clear according to the scientific literature. Since its abundance is highly dependent on a low-fat diet, the inverse correlation with plasmatic levels of cholesterol that we have found in this work could be just a direct co-linear result derived from the SD diet. Finally, butter diet shows significant differences only in one genus, *Olivibacter*, and two punctual species, *Alistipes indictintus* and *Pontibacter lucknowensis*, correlating positively with total cholesterol, ghrelin, insulin, body weight and HDL/LDL.

Lastly, within the phylum *Firmicutes*, we found pair-wise significant differences in three families (*Erysipelotrhichaceae*, *Christensenellaceae*, and *Micrococcaceae*). However, in the case of the last two families we did not find any significant correlation with any physiological variable studied. *Christensenellaceae* has been related to a lean phenotype [39], which has not been confirmed in our study since body weight shows a significant increment only in BT fed animals, which in turn have a high presence of this family, while EVOO fed animals presented the lowest body weight levels and the lowest *Christenellaceae* values as well. However, these authors [39] also highlighted this family as the most genetically heritable taxon in their study, which could partly explain our results.

A totally different case is family *Erysipelotrichaceae*, which had low global percentages but a clear and significant predominance in EVOO fed animals, although this significance is not evident in lower taxa. Family *Erysipelotrichaceae* was conformed by several species not ascribed to a specific genus that globally showed a much lower level of significance. This might indicate that they form part of a metabolic guild that shares an intestinal ecological niche. Both, family and these species altogether, correlate with total cholesterol levels. Since it has been reported that this group shows an increase after a cholic acid supplemented diet [40, 41], the increment observed could similarly be related to the amount of bile acids. Equally, a related species also predominant in EVOO fed animals, *Clostridium cocleatum*, has shown to degrade plant polyphenols [42] suggesting again the involvement of virgin olive oil polyphenols in the predominant taxa. In contrast to *Erysipelotrichaceae*, another firmicutes taxon, the genus *Fusicatenibacter* showed the highest negative correlation to cholesterol levels (p = 0.00007) in the percentage of sequences retrieved from faecal samples. As indicated above for *Prevotella*, *Fusicatenibacter* could also be affected by fat intake and metabolism. This genus belongs to clostridia cluster XVIa [43], which has been reported to have significant correlations with fecal secondary bile acids [44].

Finally, according to our study, the only firmicutes species both with significant differences (BT vs EVOO & SD) and a significant correlation with a measured parameter (water intake) is *Enterococcus gallinarum*, a lactic acid bacterium (LAB). In this respect, we would like to mention the close-to-significant increase of certain other lactic acid bacteria taxa in butter fed animal stools and decrease in EVOO. This is the case of *Lactobacillus animalis* (p = 0.051), *L*. *taiwanensis* (p = 0.051) and the genus *Lactococcus* (p = 0.066), all of them correlating positively with weight. In fact, LAB species are considered beneficial for health, GRAS (Generally Recognized As Safe) food additives [45] and widely used as probiotics [46, 47], though dissimilar correlations with weight have also been described, usually in a species or even strain-dependent manner [48].

As a summary, portrayed in Fig 8, our study shows a direct correlation between several variables involved in metabolic syndrome (blood pressure, insulin, diuresis, body weight and ghrelin) with bacterial taxa that are significantly increased in butter fed animals and decreased (except ghrelin) in the EVOO fed group. In contrast, leptin plasmatic levels negatively correlate only with taxa that are increased in the EVOO diet. Total cholesterol levels negatively correlate with the presence of two genera significantly incremented in standard diet fed animals. All these results indicate that some of the positive effects of virgin olive oil on health are significantly accompanied by parallel changes in the intestinal microbiota. However, further studies are necessary to clarify for each specific correlation whether the changes in the abundance of the bacterial taxa are a cause or a consequence of the observed physiological alterations and the mechanisms that underlie.

## Methods

### Animals

Twenty six (6 week old) male Swiss Webster ICR (CD-1) mice (Harlan Laboratories) weighing 30.1 ± 0.55 g at the beginning of the study were fed for 12 weeks a freely available standard diet (SD; standard laboratory mice diet A04, 3% fat, Panlab, Barcelona, Spain) (n = 8) or two high fat diets (35% total energy) containing either butter (n = 9) (BT; standard Panlab A04 chow supplemented with 20% butter) or extra virgin olive oil (n = 9) (EVOO; standard Panlab A04 chow supplemented with 20% extra virgin olive oil) respectively (Table 5). Extra virgin olive oil was obtained from a fully organic crop (Soler Romero, Alcaudete, Spain; 37° 33' 6.292800000012289" N, 4° 1' 35.68799999988187" W). Butter was obtained from a large commercial store (Hacendado, Mercadona, Jaén; 37° 47' 16.240199999994644" N, 3° 47' 5.314199999775182" W). Fatty acid percentage and characterization was performed (EVOO 78.6% monounsaturated fatty acids -MUFA-, 4.2% polyunsaturated fatty acids -PUFA-, 17.1% saturated fatty acids -SFA-; Butter 35.6% MUFA, 1.5% PUFA, 62.5% SFA) and EVOO polyphenol content was obtained from the producer (total polyphenol content was 527 mg/kg). All experimental procedures were performed in accordance with the European Communities Council Directive 86/609/EEC and reviewed and approved by the Bioethics Committee of the University of Jaén. Animals were individually housed in metabolic cages twenty-four hours at the end of the experimental period (twelve weeks) and food intake, water intake, diuresis, body weight (BW) and systolic blood pressure (SBP) were measured individually. In addition, faeces were also collected individually at the end of the experiment. SBP was monitored by the pleithysmographic method in un-anaesthetized animals as previously described [49]. Briefly, mice were placed in plastic holders and warmed to 37°C for each recording session. At least seven determinations were made in every session, and the mean of the stable values within a range of 5 mmHg was recorded as the SBP level. Measurements at the beginning and end were discarded. At the end of the experimental period, after collecting faeces and after recording food and water intake, diuresis, body weight and SBP, blood samples were obtained from the left cardiac ventricle under equithensin anaesthesia (2 ml/kg body weight) (42.5 g/l chloralhydrate dissolved in 19.76 ml ethanol, 9.72 g/l Nembutal, 0.396 l/l propylenglycol and 21.3 g/l magnesium sulphate in distilled water) and then animals were sacrificed being perfused with saline solution through the left cardiac ventricle. Plasma was isolated by centrifugation of blood samples for 10 min at 2000 g, using heparin as anticoagulant, and stored at -20°C. In plasma samples, insulin, fasting glucose, triglycerides, total cholesterol and HDL were determined as previously reported [49, 50]. Plasma concentrations of leptin and ghrelin, were measured in a multianalyte profiling by using the Luminex-100 system and the XY Platform [51]. All analyses were performed according to the manufacturers' protocols. Statistical significance of the different values was checked by ANOVA or, alternatively, with Kruskal-Wallis test whenever the ANOVA hypotheses were not met.

**Table 5:** Nutrient composition and energy content of standard and high fat diets enriched with extra virgin olive oil and butter.

Diet	SD		EVOO		BT	
	g/100 g	% energy	g/100 g	% energy	g/100 g	% energy
**Protein**	16.5	20	16.5	14	16.5	14
**Carbohydrates**	60	72	55	48	55	48
**Fat**	3	8	20	38	20	38
**Total energy (kJ/g)**	14.2		19.6		19.6	

### Microbial biodiversity

In order to study the bacterial composition in faeces, DNA was extracted using QIAamp DNA Stool Kit. Twenty six DNA samples, corresponding to eight faecal samples from eight mice under a standard diet and eighteen faecal samples from nine mice fed high butter and nine fed a high extra virgin olive oil diet, were pyrosequenced at Lifesequencing (Valencia, Spain) using Roche GS-FLX-Titanium + 454 pyrosequencing technology, targeting the 16S ribosomal DNA V3–V4 region (V3fwd: 5’ TCCGTCAATTYMTTTRAGT 3’, V4rev: 5’ CCTACGGGAGGCAGCAG 3’). Thermal cycling consisted of initial denaturation at 94°C for 2 minutes followed by 30 cycles of denaturation at 94°C for 20 seconds, annealing at 50°C for 30 seconds, and extension at 72°C for 5 minutes. Twenty-six libraries were constructed, the different amplifications were individually measured and the quantity of amplified DNA was estimated using Quant-iTTM PicoGreen (Invitrogen). The 26 amplified samples were pooled into one single equimolar pool that was used in the sequencing step. A quality filter was applied to the sequences in order to delete those with poor quality. Those bases in extreme positions that did not have Q20 or more phred score were removed, and later, sequences whose quality mean did not reach the Q20 threshold as a mean were also deleted. The rest of the sequences obtained were trimmed for adaptors and PCR primer removal, using “cutadapt v. 1.4.1”, and binned for a minimal sequence length of 300 bases. All further remaining sequences were checked for quimeras using the UCHIME v. 4.2.40 program. The resulting sequences were assigned to different taxonomic levels using the Ribosomal Database Project Classifier. Rarefaction curves were obtained for each sample and taxonomical levels were analysed in order to confirm they had reached the *plato* and no more taxonomical groups were expected to be found if sequencing were increased.

For the statistical analysis, as the ANOVA assumptions are not met to test equality of the distributions according to the different types of diet we used the Kruskal-Wallis test. A significance level of 0.05 has been chosen. Provided that significant differences were detected we applied post-hoc tests for pairwise multiple comparisons (all computations were made using SPSS and R). Also, those distributions that show statistically significant differences among the diets were used as independent variables in multiple linear regression models where the physiological variables considered were the dependent variables (all regression models were fitted using the open-source statistical package Gretl). The approach used to fit the regression model has been a stepwise regression by backward elimination, so at a starting point all independent variables were included in the model, in the following steps the deletion of each variable was decided using p-values as model comparison criterion, deleting the variable (if any) that improved the model the most by being deleted, and repeating this process until no further improvement was possible. Richness and diversity indexes were carried out in QIIME [52].

## Supporting information

S1 Table Kruskal-Wallis sum of ranks for each type of diet, p-value of K-W test, and pairwise comparisons p-values of the families with significant differences between diets in the percentage of sequences retrieved from faecal samples. (DOCX)

S2 Table Kruskal-Wallis sum of ranks for each type of diet, p-value of K-W test, and pairwise comparisons p-values of the genera with significant differences between diets in the percentage of sequences retrieved from faecal samples. (DOCX)

S3 Table Kruskal-Wallis sum of ranks for each type of diet, p-value of K-W test, and pairwise comparisons p-values of the species with significant differences between diets in the percentage of sequences retrieved from faecal samples. (DOCX)

S1 File Minimal underlying data supporting Table 1. (XLSX)
