# Supplementary material for: Influence of a diet enriched with virgin olive oil or butter on mouse gut microbiota and its correlation to physiological and biochemical parameters related to metabolic syndrome
Source: PLoS One. 2018 Jan 2;13(1):e0190368. doi: 10.1371/journal.pone.0190368 (PMC5749780; doi:10.1371/journal.pone.0190368)
Supplement: S1 Table — (DOCX) [file pone.0190368.s001.docx]

| **Family** | **SD** | **EVOO** | **BT** | **Significance** | **Pairwise significance level** |
| --- | --- | --- | --- | --- | --- |
| *Prevotellaceae* | 19.50 | 9.22 | 12.44 | 0.019 | EVOO vs BT 1.000  **EVOO vs SD 0.017**  BT vs SD 0.173 |
| *Desulfovibrionaceae* | 11.62 | 10.00 | 18.67 | 0.039 | **EVOO vs BT 0.049**  EVOO vs SD 1.000  BT vs SD 0.174 |
| *Marinilabiliaceae* | 20.62 | 11.33 | 9.33 | 0.006 | EVOO vs BT 1.000  **EVOO vs SD 0.037**  **BT vs SD 0.007** |
| *Erysipelotrichaceae* | 8.12 | 19.56 | 12.22 | 0.007 | EVOO vs BT 0.126  **EVOO vs SD 0.006**  BT vs SD 0.811 |
| *Sutterellaceae* | 7.56 | 18.11 | 14.17 | 0.017 | EVOO vs BT 0.822  **EVOO vs SD 0.014**  BT vs SD 0.227 |
| *Eubacteriaceae* | 8.00 | 15.28 | 16.61 | 0.047 | EVOO vs BT 1.000  EVOO vs SD 0.150  BT vs SD 0.061 |
| *Christensenellaceae* | 16.75 | 7.89 | 16.22 | 0.019 | EVOO vs BT 0.052  **EVOO vs SD 0.042**  BT vs SD 1.000 |
| *Microbacteriaceae* | 11.00 | 11.00 | 18.22 | 0.004 | **EVOO vs BT 0.011**  EVOO vs SD 1.000  **BT vs SD 0.014** |
