# Supplementary material for: Influence of a diet enriched with virgin olive oil or butter on mouse gut microbiota and its correlation to physiological and biochemical parameters related to metabolic syndrome
Source: PLoS One. 2018 Jan 2;13(1):e0190368. doi: 10.1371/journal.pone.0190368 (PMC5749780; doi:10.1371/journal.pone.0190368)
Supplement: S2 Table — (DOCX) [file pone.0190368.s002.docx]

| **Genera** | **SD** | **EVOO** | **BT** | **Significance** | **Pairwise significance level** |
| --- | --- | --- | --- | --- | --- |
| *Prevotella* | 19.12 | 9.00 | 13.00 | 0.024 | EVOO vs BT 0.802  **EVOO vs SD 0.019**  BT vs SD 0.298 |
| *Marvinbryantia* | 16.22 | 8.44 | 16.22 | 0.049 | EVOO vs BT 0.093  EVOO vs SD 0.116  BT vs SD 1.000 |
| *Desulfovibrio* | 12.88 | 8.94 | 18.61 | 0.026 | **EVOO vs BT 0.022**  EVOO vs SD 0.870  BT vs SD 0.368 |
| *Anaerophaga* | 21.62 | 9.56 | 10.22 | 0.001 | EVOO vs BT 1.000  **EVOO vs SD 0.003**  **BT vs SD 0.006** |
| *Fusicatenibacter* | 19.56 | 9.56 | 12.06 | 0.021 | EVOO vs BT 1.000  **EVOO vs SD 0.021**  BT vs SD 0.130 |
| *Parasutterella* | 7.56 | 18.11 | 14.17 | 0.017 | EVOO vs BT 0.822  **EVOO vs SD 0.014**  BT vs SD 0.227 |
| *Eubacterium* | 8.00 | 15.39 | 16.50 | 0.048 | EVOO vs BT 1.000  EVOO vs SD 0.140  BT vs SD 0.066 |
| *Christensenella* | 16.75 | 7.89 | 16.22 | 0.019 | EVOO vs BT 0.052  **EVOO vs SD 0.042**  BT vs SD 1.000 |
| *Erysipelotrichaceae; null* | 7.94 | 16.78 | 16.17 | 0.047 | EVOO vs BT 1.000  EVOO vs SD 0.104  BT vs SD 0.080 |
| *Olivibacter* | 8.62 | 11.86 | 19.44 | 0.011 | EVOO vs BT 0.108  EVOO vs SD 1.000  **BT vs SD 0.011** |
| *Marispirillum* | 7.38 | 16.17 | 16.28 | 0.022 | EVOO vs BT 1.000  **EVOO vs SD 0.049**  **BT vs SD 0.045** |
| *Marinilabilia* | 7.69 | 18.33 | 13.83 | 0.010 | EVOO vs BT 0.566  **EVOO vs SD 0.008**  BT vs SD 0.245 |
| *Curtobacterium* | 11.0 | 11.0 | 18.22 | 0.004 | **EVOO vs BT 0.011**  EVOO vs SD 1.000  **BT vs SD 0.014** |
| *Enterobacter* | 12.0 | 12.0 | 16.33 | 0.047 | EVOO vs BT 0.091  EVOO vs SD 1.000  BT vs SD 0.107 |
| *Pantoea* | 12.0 | 12.0 | 16.33 | 0.047 | EVOO vs BT 0.091  EVOO vs SD 1.000  BT vs SD 0.107 |
