# Supplementary material for: Influence of a diet enriched with virgin olive oil or butter on mouse gut microbiota and its correlation to physiological and biochemical parameters related to metabolic syndrome
Source: PLoS One. 2018 Jan 2;13(1):e0190368. doi: 10.1371/journal.pone.0190368 (PMC5749780; doi:10.1371/journal.pone.0190368)
Supplement: S3 Table — (DOCX) [file pone.0190368.s003.docx]

| **Species** | **SD** | **EVOO** | **BT** | **Significance** | **Pairwise significance level** |
| --- | --- | --- | --- | --- | --- |
| *Clostridium celerecrescens* | 20.12 | 13.22 | 7.89 | 0.004 | EVOO vs BT 0.417  EVOO vs SD 0.190  **BT vs SD 0.003** |
| *Bacteroides fragilis* | 10.38 | 20.22 | 9.56 | 0.005 | **EVOO vs BT 0.009**  **EVOO vs SD 0.024**  BT vs SD 1.000 |
| *Marvinbryantia formatexigens* | 16.12 | 8.44 | 16.22 | 0.049 | EVOO vs BT 0.093  EVOO vs SD 0.116  BT vs SD 1.000 |
| *Desulfovibrio desulfuricans* | 13.80 | 8.94 | 18.50 | 0.029 | **EVOO vs BT 0.024**  EVOO vs SD 0.825  BT vs SD 0.416 |
| *Anaerophaga thermohalophila* | 21.62 | 9.56 | 10.22 | 0.001 | EVOO vs BT 1.000  **EVOO vs SD 0.003**  **BT vs SD 0.006** |
| *Parapedobacter composti* | 17.25 | 7.89 | 15.78 | 0.023 | EVOO vs BT 0.086  EVOO vs SD 0.035  BT vs SD 1.000 |
| *Fusicatenibacter saccharivorans* | 19.56 | 9.56 | 12.06 | 0.021 | EVOO vs BT 1.000  **EVOO vs SD 0.021**  BT vs SD 0.130 |
| *Anaerostipes butyraticus* | 12.25 | 9.44 | 18.67 | 0.033 | **EVOO vs BT 0.032**  EVOO vs SD 1.000  BT vs SD 0.253 |
| *Parabacteroides merdae* | 20.62 | 6.72 | 13.94 | 0.001 | EVOO vs BT 0.131  **EVOO vs SD 0.001**  BT vs SD 0.211 |
| *Clostridium cellulolyticum* | 8.06 | 14.50 | 17.33 | 0.040 | EVOO vs BT 1.000  EVOO vs SD 0.250  **BT vs SD 0.038** |
| *Alistipes indistinctus* | 12.75 | 9.22 | 18.44 | 0.035 | **EVOO vs BT 0.031**  EVOO vs SD 1.000  BT vs SD 0.372 |
| *Parasutterella excrementihominis* | 7.56 | 18.11 | 14.17 | 0.017 | EVOO vs BT 0.822  **EVOO vs SD 0.014**  BT vs SD 0.227 |
| *Christensenella minuta* | 16.75 | 7.89 | 16.22 | 0.019 | EVOO vs BT 0.052  **EVOO vs SD 0.042**  BT vs SD 1.000 |
| *Ruminococcus flavefaciens* | 16.81 | 9.50 | 14.56 | 0.045 | EVOO vs BT 0.259  **EVOO vs SD 0.048**  BT vs SD 1.000 |
| *Dysgonomonas mossii* | 19.75 | 11.28 | 10.17 | 0.019 | EVOO vs BT 1.000  EVOO vs SD 0.064  **BT vs SD 0.027** |
| *Paraprevotella clara* | 17.62 | 8.56 | 14.78 | 0.041 | EVOO vs BT 0.250  **EVOO vs SD 0.043**  BT vs SD 1.000 |
| *Olivibacter sitiensis* | 8.19 | 12.28 | 19.44 | 0.008 | EVOO vs BT 0.139  EVOO vs SD 0.811  **BT vs SD 0.007** |
| *Marispirillum indicum* | 7.38 | 16.17 | 16.28 | 0.022 | EVOO vs BT 1.000  **EVOO vs SD 0.049**  **BT vs SD 0.045** |
| *Prevotella buccalis* | 17.50 | 10.50 | 12.94 | 0.036 | EVOO vs BT 1.000  **EVOO vs SD 0.032**  BT vs SD 0.291 |
| *Prevotella dentalis* | 17.75 | 11.00 | 12.22 | 0.020 | EVOO vs BT 1.000  **EVOO vs SD 0.025**  **BT vs SD** 0.092 |
| *Clostridium clariflavum* | 10.25 | 11.00 | 18.89 | 0.030 | EVOO vs BT 0.081  EVOO vs SD 1.000  BT vs SD 0.057 |
| *Tyzzerella nexilis* | 16.88 | 12.00 | 12.00 | 0.026 | EVOO vs BT 1.000  EVOO vs SD 0.054  BT vs SD 0.054 |
| *Clostridium cocleatum* | 11.75 | 18.44 | 10.11 | 0.016 | **EVOO vs BT 0.019**  EVOO vs SD 0.102  BT vs SD 1.000 |
| *Eubacterium coprostanoligenes* | 7.88 | 13.83 | 18.17 | 0.020 | EVOO vs BT 0.675  EVOO vs SD 0.316  **BT vs SD 0.015** |
| *Eubacterium tortuosum* | 9.62 | 18.39 | 12.06 | 0.024 | EVOO vs BT 0.152  **EVOO vs SD 0.026**  BT vs SD 1.000 |
| *Parapedobacter koreensis* | 8.38 | 18.44 | 13.11 | 0.021 | EVOO vs BT 0.393  **EVOO vs SD 0.017**  BT vs SD 0.579 |
| *Lactobacillus apodemi* | 8.25 | 13.11 | 18.56 | 0.015 | EVOO vs BT 0.349  EVOO vs SD 0.521  **BT vs SD 0.012** |
| *Spiroplasma velocicrescens* | 8.25 | 12.94 | 18.72 | 0.013 | EVOO vs BT 0.287  EVOO vs SD 0.567  **BT vs SD 0.010** |
| *Marinilabilia salmonicolor* | 7.69 | 13.83 | 18.33 | 0.010 | EVOO vs BT 0.566  **EVOO vs SD 0.008**  BT vs SD 0.245 |
| *Anaeroplasma abactoclasticum* | 7.25 | 16.22 | 16.33 | 0.015 | EVOO vs BT 1.000  **EVOO vs SD 0.036**  **BT vs SD 0.033** |
| *Pontibacter lucknowensis* | 10.44 | 9.67 | 20.06 | 0.001 | **EVOO vs BT 0.003**  EVOO vs SD 1.000  **BT vs SD 0.009** |
| *Clostridium colicanis* | 12.00 | 12.00 | 16.33 | 0.048 | EVOO vs BT 0.091  EVOO vs SD 1.000  BT vs SD 0.107 |
| *Staphylococcus xylosus* | 11.00 | 12.00 | 17.00 | 0.043 | EVOO vs BT 0.162  EVOO vs SD 1.000  BT vs SD 0.057 |
| *Enterococcus gallinarum* | 11.00 | 11.00 | 18.22 | 0.004 | **EVOO vs BT 0.011**  EVOO vs SD 1.000  **BT vs SD 0.014** |
| *Enterococcus faecalis* | 10.50 | 11.78 | 17.89 | 0.014 | EVOO vs BT 0.065  EVOO vs SD 0.065  **BT vs SD 0.021** |
| *Curtobacterium plantarum* | 11.00 | 11.00 | 18.22 | 0.004 | **EVOO vs BT 0.011**  EVOO vs SD 1.000  **BT vs SD 0.014** |
| *Enterobacter asburiae* | 12.00 | 12.00 | 16.33 | 0.047 | EVOO vs BT 0.091  EVOO vs SD 1.000/1.000  BT vs SD 0.107 |
| *Pantoea eucalypti* | 12.00 | 12.00 | 16.33 | 0.047 | EVOO vs BT 0.091  EVOO vs SD 1.000  BT vs SD 0.107 |
| *Desulfovibrio litoralis* | 12.00 | 12.00 | 16.33 | 0.047 | EVOO vs BT 0.091  EVOO vs SD 1.000  BT vs SD 0.107 |
| *Mucilaginibacter daejeonensis* | 11.00 | 18.22 | 11.00 | 0.007 | **EVOO vs BT 0.011**  **EVOO vs SD 0.014**  BT vs SD 1.000 |
| *Staphylococcus aureus* | 12.00 | 16.33 | 12.00 | 0.047 | EVOO vs BT 0.091  EVOO vs SD 0.107  BT vs SD 1.000 |
| *Clostridium beijerinckii* | 12.00 | 16.33 | 12.00 | 0.047 | EVOO vs BT 0.091  EVOO vs SD 0.107  BT vs SD 1.000 |
| *Clostridium frigidicarnis* | 12.00 | 16.33 | 12.00 | 0.048 | EVOO vs BT 0.091  EVOO vs SD 0.107  BT vs SD 1.000 |
